# Levels of anti-citrullinated protein antibodies and IgM rheumatoid factor are not associated with outcome in early arthritis patients: a cohort study

**DOI:** 10.1186/ar2907

**Published:** 2010-01-12

**Authors:** Jennie Ursum, Wouter H Bos, Nancy van Dillen, Ben AC Dijkmans, Dirkjan van Schaardenburg

**Affiliations:** 1 Jan van Breemen Institute, department of rheumatology, Dr. Jan van Breemenstraat 2, 1056 AB Amsterdam, The Netherlands; 2 VU University Medical Centre, department of rheumatology, Postbus 7057, 1007 MB Amsterdam, The Netherlands

## Abstract

**Introduction:**

To investigate whether baseline levels of anti-citrullinated protein antibody (ACPA) or IgM rheumatoid factor (IgM-RF) and changes in the year thereafter are associated with disease activity, functional and radiographic outcome in early arthritis patients, and provide additional information over baseline autoantibody status.

**Methods:**

In 545 early arthritis patients ACPA and IgM-RF levels, disease activity (DAS28), the Health Assessment Questionnaire (HAQ) and Sharp/Van der Heijde Score (SHS) were assessed annually. Baseline status, levels and first-year changes of the autoantibodies were associated with these measures at the two-year follow-up and sub-analysed according to autoantibody status.

**Results:**

The mean age was 52.7 years, 69% was female, at baseline 56% was ACPA positive, 47% IgM-RF positive. At the two-year follow-up the mean DAS28 was 2.88, and the median HAQ and SHS were 0.38 and 1, respectively. At one year, ACPA and IgM-RF levels had decreased by 31% and 56%, respectively. A switch from negative to positive occurred in 2% for ACPA and 3% for IgM-RF. Positive ACPA and RF status were both associated with SHS at two years (*P *< 0.001), but baseline levels only showed a minor correlation of ACPA with DAS28 and HAQ at two years. Level changes were not associated with the outcome parameters.

**Conclusions:**

Baseline levels and first-year changes of ACPA and IgM-RF are hardly associated with outcome after two years. Seroconversion seldom occurs. Therefore, it does not appear useful to repeat ACPA or IgM-RF measurements.

## Introduction

Rheumatoid arthritis (RA) is often accompanied by autoimmune phenomena, notably anti-citrullinated protein antibodies (ACPA) and rheumatoid factor (RF). Although ACPA-positive RA cannot be distinguished from ACPA-negative RA at first presentation [1,2], several studies have demonstrated that the presence of ACPA is prognostic for disease severity, radiographic erosions, as well as the development of RA in synovitis of recent onset [1,3-8]. Recently, higher ACPA levels have been found in patients who developed RA compared with those who did not develop RA [9]. Most studies assessed the predictive value of the presence of ACPA [3,8,10-15]. However, it is as yet unclear whether high levels of ACPA predict poorer outcome [16-20]. In a prospective study of 104 early RA patients, higher baseline ACPA levels were associated with erosive disease after two years [20]. Another study of 99 early RA patients reported a small, almost significant correlation between baseline serum ACPA levels and radiographic progression after five years [18]. A third study of 238 early RA patients found a higher radiographic progression rate after 10 years of high-positive ACPA versus low-positive ACPA patient groups [19].

Two studies assessed levels of ACPA in patients with longstanding RA. One of these reported a weak association (in 180 patients) between ACPA levels and radiographic progression rate [16]. The other was a cross-sectional study of 241 RA patients with a mean disease duration of 8.6 years, in which mean ACPA levels were similar in patients with or without erosions [17].

RF, mostly measured as immunoglobulin (Ig)M-RF, is still widely used as a serological marker for the diagnosis of RA, although it is also frequently observed in other inflammatory diseases [21] and in healthy elderly persons [22] suggesting that RF can be a consequence of nonspecific immune activation. Its presence is a prognostic marker of disease activity and erosive disease [10,20]. Higher IgM-RF levels have been associated with a higher risk for the development of RA[23]. IgM-RF levels also seem to be associated with future radiographic damage: in three studies, in which 78 to 149 early RA patients participated, a correlation was found between baseline IgM-RF levels and radiographic damage after two to three years [20,24,25].

Reports of ACPA or IgM-RF levels and outcome in early arthritis are therefore still few and to our knowledge no data are available on changes in levels of ACPA or IgM-RF as a predictor of disease outcome. Changes in autoantibody levels could possibly serve as markers of response to therapy and thus be related to outcome. Therefore, we investigated whether baseline status or levels of ACPA or IgM-RF and their changes in the year thereafter are associated with disease activity, functional and radiographic outcome in a large group of early arthritis patients, and whether analysis of levels provides additional information over baseline antibody status.

## Materials and methods

The early arthritis cohort at the Jan van Breemen Institute, a large rheumatology clinic in Amsterdam, has been described previously [26]. The cohort consists of patients aged older than 18 years with peripheral arthritis of two or more joints and a symptom duration of less than three years, who were referred from 1995 onwards. Patients who were previously treated with a disease modifying anti-rheumatic drug (DMARD) and those with spondylarthropathy, reactive arthritis, crystal-induced arthropathy, systemic lupus erythematosus, Sjögren's syndrome, or osteoarthritis were excluded. The study was approved by the local medical ethics committee and all patients gave written informed consent to be included in the study. For the present analysis, all patients with available ACPA and IgM-RF data at baseline and after one year, and available outcome measures at two-years follow up were included.

### Antibody measurements

ACPA levels were measured as anti-cyclic citrullinated peptide antibodies (second generation anti-CCP ELISA, Axis Shield, Dundee, UK). The anti-CCP test was performed according to the instructions of the manufacturer with a cut-off level for positivity set at 5 Arbitrary Units (AU)/ml. The day to day variation (CV) was 7.4% (n = 98). Anti-CCP levels in sera reaching 1000 AU were not further diluted. IgM-RF was measured by in-house ELISA as described previously [27]. The cut-off level for IgM-RF antibody positivity is set at 30 IU determined on the basis of receiver operator characteristics (ROC) curves described previously [27].

### Outcome measures

Disease activity was assessed with the Disease Activity Score in 28 joints (DAS28) [28]. Functional status was measured by the validated Dutch version of the Health Assessment Questionnaire (HAQ) [29]. Radiographic damage was assessed with the Sharp/Van der Heijde Score (SHS) by one experienced rheumatologist, who was blinded to the other variables. Two rheumatologists, with an intraclass correlation coefficient of 0.95, each performed part of the scoring.

### Analysis

The baseline characteristics were age, sex, symptom duration, percentage of patients who fulfilled the American College of Rheumatology (ACR) criteria for RA and the percentage IgM-RF and ACPA positivity were compared between those patients included in the study (n = 545) and those excluded (n = 1309). The change in ACPA level was calculated as an absolute change and as a relative change compared with baseline. Outcome measures at the two-year follow up were DAS28, HAQ and SHS, all used as continuous variables.

Differences between groups with positive or negative autoantibody status were tested using Mann-Whitney U test or the chi-squared test. Correlations were determined by Pearson rho or Spearman rho as appropriate. Partial correlations were used to correct for baseline values. For an association between change in ACPA level and dichotomous variables, logistic regression analysis was used. Subanalyses were performed for positive and negative autoantibody status at baseline. All analyses were performed using SPSS version 16.0 (SPSS Institute Inc., Cary, NC, USA).

## Results

### Patient characteristics

In the study, 545 patients were included, with a mean age of 53 years, 69% was female. Sixty-three percent fulfilled the ACR criteria for RA at baseline or after one year. At baseline, 56% was ACPA positive and 47% was IgM-RF positive. At the two-year follow up the mean (standard deviation) DAS28 was 2.88 (1.27), the median (interquartile range (IQR)) HAQ was 0.38 (0 to 0.8) and the median (IQR) SHS was 1 (0 to 6). At two years the median (IQR) number of DMARD used was 2 (1 to 3); 27% of the patients had used hydroxychloroquine, 39% sulphasalazine, 80% methotrexate and 25% prednisone, sometimes in combination, while 2% had not used any DMARD.

### Autoantibodies: change in status and levels between baseline and one year

After one year, median ACPA levels had decreased significantly to 78% (*P *< 0.001) of the baseline levels. Higher baseline levels were correlated with a larger absolute change in the first year (r = -0.44, *P *< 0.001), but barely with the relative change (r = -0.09, *P *= 0.04). The decrease was mainly caused by ACPA-positive patients; in this subgroup the median (IQR) ACPA level decreased significantly, equalling 69% (40 to 114%) of the baseline value (Table 1). Of the ACPA-positive patients at baseline, 4% became negative for ACPA after one year. In ACPA-negative patients at baseline only 2% became positive for ACPA after one year follow up.

**Table 1:** The relation between ACPA status and levels and outcome variables

	Correlation of ACPA levels at baseline	*P* ^1^	ACPA positive at baseline (n = 304)	ACPA negative at baseline (n = 241)	*P* ^2^
ACPA level at baseline (AU/ml), median (IQR)	x		89 (27-214)	1 (0-2)	
ACPA level at 1 year (AU/ml), median (IQR)	x		63 (22-156)	1 (0-2)	
DAS28 at baseline	0.09	0.04	4.81 (1.78)	4.74 (1.36)	0.42
HAQ at baseline	-0.1	0.83	0.94 (0.5-1.5)	1 (0.5-1.62)	0.31
SHS at baseline	0.12	0.01	0 (0-2)	0 (0-1)	0.01
DAS28 at 2-year follow up	0.06	0.16	2.91 (1.28)^&^	2.84 (1.26)^&^	0.78
HAQ at 2-year follow up	0.05	0.24	0.44 (0-1)	0.38 (0-0.88)	0.72
SHS at 2-year follow up	0.20	0.00	2 (0-9)	0 (0-3)	0.00

^#^Spearman correlation ^$^Median with IQR unless stated otherwise^&^Mean with SD *P*-value of correlation^2 ^*P*-value ACPA positive versus ACPA negative.

ACPA = anti-citrullinated protein antibody; DAS = disease activity score; HAQ = Health Assessment Questionnaire; IQR = interquartile range; SD = standard deviation; SHS = Sharp-van der Heijde score.

Median IgM-RF levels had decreased after one year to 72% (*P *< 0.001) of the baseline levels. Higher baseline levels were associated with larger absolute as well as relative changes (r = -0.71 and -0.54, respectively, both *P *< 0.001). Also this decrease was mainly caused by IgM-RF-positive patients; in this subgroup the median (IQR) IgM-RF level decreased significantly, equalling 44% (27 to 71%) of the baseline value (Table 1). Of the IgM-RF-positive patients at baseline, 35% became negative for IgM-RF after one year of follow up. In IgM-RF-negative patients, 3% became positive for IgM-RF after one year of follow up.

### Baseline autoantibody levels and status, and outcome

#### Outcome parameters at baseline

ACPA and IgM-RF levels were modestly correlated with SHS (r = 0.12 and r = 0.11, respectively, both *P *= 0.01) and not with DAS28 and HAQ (Tables 1 and 2). Patients positive for ACPA or IgM-RF had at median more radiographic damage at baseline compared with those negative for ACPA of IgM-RF (Tables 1 and 2).

**Table 2:** The relation between IgM-RF status and levels and outcome variables

	Correlation of IgM-RF levels at baseline^#^	*P* ^1^	IgM-RF positive at baseline (n = 255)^$^	IgM-RF negative at baseline (n = 280)^$^	*P* ^2^
IgM-RF level at baseline (IU/ml), median (IQR)	x		100 (60-200)	10 (6-14)	
IgM-RF level at 1 year (IU/ml), median (IQR)	x		51 (23-97)	9 (5-10)	
DAS28 at baseline	0.11	0.01	4.88 (1.19)	1.66 (1.29)	0.01
HAQ at baseline	0.01	0.79	1 (0.5-1)	0.94 (0.5-1.5)	0.49
SHS at baseline	0.11	0.02	0 (0-1)	0 (0-0)	0.00
DAS28 at 2-year follow-up	0.17	0.69	2.92 (1.30)^&^	2.84 (1.23)^&^	0.58
HAQ at 2-year follow-up	0.04	0.41	0.5 (0-0.88)	0.38 (0-0.88)	0.43
SHS at 2-year follow-up	0.22	0.00	2 (0-9)	0 (0-4)	0.00

^#^Spearman correlation ^$^Median with IQR unless stated otherwise^&^Mean with SD *P*-value of correlation^2 ^*P*-value IgM-RF positive versus IgM-RF negative.

DAS = disease activity score; HAQ = Health Assessment Questionnaire; Ig = immunoglobulin; IQR = interquartile range; RF = rheumatoid factor; SD = standard deviation; SHS = Sharp-van der Heijde score.

#### Outcome parameters at two years

Baseline ACPA and IgM-RF levels were not correlated with DAS28 or HAQ at the two-year follow up. Levels were only correlated with SHS (r = 0.2 and r = 0.22, respectively, both *P *= 0.01). When correcting for baseline SHS only the correlation between IgM-RF levels and SHS remained (r = 0.14, *P *= 0.002).

Patients positive for ACPA or IgM-RF at baseline had at median more radiographic damage at the two-year follow up compared with those negative for ACPA of IgM-RF at baseline (Tables 1 and 2). However, mean DAS28 and median HAQ at the two-year follow up did not differ between patients with positive or negative autoantibody status.

In the subgroup of ACPA-positive patients, baseline ACPA levels were modestly correlated only with DAS28 and HAQ at the two-year follow up (r = 0.15 and r = 0.13, respectively, both *P *< 0.05), while no correlation was found with SHS (r = -0.04, *P *= 0.54). When correcting for baseline DAS or baseline HAQ, these correlations were no longer present.

In the subgroup of patients with a positive IgM-RF status at baseline there was no correlation between IgM-RF levels and SHS (r = 0.05, *P *= 0.38).

### Change in autoantibody levels and outcome

#### First-year change in levels and outcome at two year follow-up

**Figure 1:**
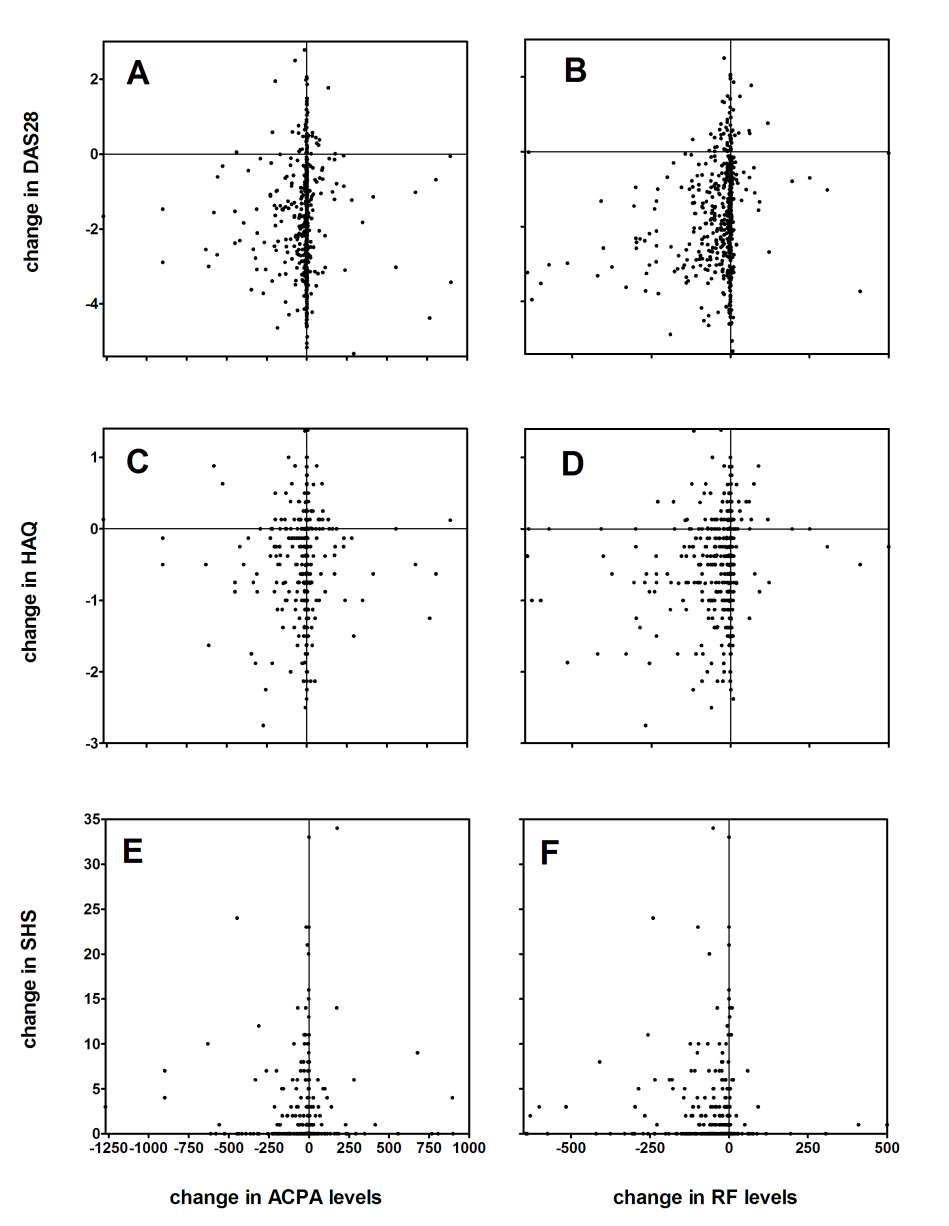
**Absolute change of IgM-RF and ACPA compared with the absolute change of DAS28, HAQ and SHS**. The x-axes of a, c and f represent change in anti-citrullinated protein antibody (ACPA) levels in the first year. The x-axes of b, d and e represent the change in IgM-rheumatoid factor (RF) levels in the first year. Negative numbers indicate a decrease in levels whereas a positive number indicates an increase in levels. The Y-axes of a and b represent the change in the Disease Activity Score on 28 joints (DAS28) in the first year. The Y-axes of c and d represent the change in the Health Assessment Score (HAQ) in the first year. The Y-axes of e and f represent the change in the Sharp/van der Heijde Score (SHS) in the first year. Negative numbers indicate a decrease in the score and a positive number an increase, though for the SHS only an increase in score is possible.

Absolute or relative changes in ACPA level in the first year were not correlated to DAS28, HAQ or SHS at the two-year follow up (all *P *> 0.13; Figure 1). First-year changes in IgM-RF level were not correlated to DAS28 and HAQ (Figure 1), but absolute and relative changes were modestly correlated to SHS at two years (r = -0.17 vs. R = -0.15, both *P *< 0.001). In ACPA or IgM-RF-positive patients, absolute as well as relative changes in levels in the first year were not correlated with the two-year outcome measures (all *P *> 0.10). ACPA or IgM-RF-negative patients were not further analysed since their levels did not change significantly.

## Discussion

The well-known association between ACPA or RF status and radiographic outcome was confirmed in the present study. Therefore, we focussed on ACPA and IgM-RF levels at baseline and changes in the year thereafter in relation to outcome at two years, to investigate whether this would provide additional prognostic information in early arthritis patients.

The results were that baseline levels were only marginally associated with later radiographic damage. The first-year change in IgM-RF levels was modestly associated with SHS at the two-year follow up; however, this association was lost in the subgroup of patients with a positive baseline status.

It was also noted that ACPA and RF rarely switched from negative to positive after one year: in 2 and 3%, respectively. On the other hand, decreases in levels over the first year occurred in the majority of patients, with a median decrease in level of 22% for ACPA and 28% for RF. Recently, another study in very early RA patients reported an increase of ACPA levels of 10% and a decrease of IgM-RF levels of 14% [30]. The marked decrease found in the present study is probably due to treatment, because it is similar to what has been found in reaction to TNF-blocking therapy in established RA [31]. The differential decrease supports the notion that RF is more a disease activity marker and ACPA more a disease-specific marker [31,32].

At baseline, ACPA levels and status were associated only with SHS at baseline, while IgM-RF levels and status were associated with DAS28 and SHS at baseline, as was also reported previously [20,30]. For the two-year outcome, there was only a correlation between baseline autoantibody levels and SHS, as had been found before [18,20,24,30], although most studies [3,8,10-15] only addressed autoantibody status. High autoantibody levels at baseline seem to be modestly associated with future radiographic damage. However, in the present study these associations were lost when stratifying for autoantibody status. Previous studies did not analyse separately according to autoantibody status. Therefore, their results could be a reflection of autoantibody status.

The present results show that changes in ACPA level during the first year of follow up are not correlated to the outcome at two years, while there was a weak but negative correlation in the first-year change in IgM-RF levels and SHS at two years. One earlier study reported the absence of a relation between changes in antibody levels and changes in disease activity in the same time period [2]. We are not aware of any study reporting changes in levels as a predictor for outcome later in the disease course.

## Conclusions

In conclusion, measurement of ACPA or RF levels in the first year in early arthritis patients does not provide additional information over autoantibody status at baseline in the prediction of the outcome after two years. As seroconversion of autoantibody status after one year is rare, except for downward seroconversion of IgM-RF, it does not seem useful in general to repeat these tests. It is more the presence than the levels of the autoantibodies that counts.

## Abbreviations

ACPA: anti-citrullinated protein antibody; ACR: American College of Rheumatology; AU: arbitrary units; CCP: cyclic citrullinated peptide; DAS28: Disease Activity Score on 28 joints; DMARD: disease modifying anti-rheumatic drugs; ELISA: enzyme-linked immunosorbent assay; HAQ: Health Assessment Score; Ig: immunoglobulin; IQR: interquartile range; RA: rheumatoid arthritis; RF: rheumatoid factor; ROC: receiver operator characteristic; SHS: Sharp/Van der Heijde Score; TNF: tumor necrosis factor.

## Competing interests

The authors declare that they have no competing interests.

## Authors' contributions

JU performed analysis and interpretation of the data and drafted the manuscript. WHB contributed to study design, interpretation of the data and drafting of the manuscript. ND contributed to acquisition of the data and drafting the manuscript. BD helped designing the study and drafting the manuscript and DvS contributed to study design, interpretation of the data and drafting of the manuscript. All authors read and approved the final manuscript.
